# VOCs Turn Up in Well Water: Sensitive Measure Reveals Groundwater Contaminant

**DOI:** 10.1289/ehp.115-a550b

**Published:** 2007-11

**Authors:** Tina Adler

About 15% of the U.S. population get their drinking and household water from a largely unmonitored source: private residential wells. About 400,000 new wells are drilled every year. A new USGS study suggests that the water in a small percentage of domestic wells could contain unsafe levels of volatile organic compounds (VOCs) **[*EHP* 115:1539–1546; Rowe et al.]**.

VOCs come from a wide variety of sources, including gasoline, plastics, paints, dyes, solvents, adhesives, insecticides, and spot removers, and have wide-ranging health effects. The chemical and physical properties of VOCs allow the compounds to move between the atmosphere, soil, surface water, and groundwater. Once in the environment, some VOCs degrade quickly whereas others persist for decades.

The USGS collected data on 55 VOCs primarily between 1991 and 2002. The team analyzed water samples before homeowners treated or filtered the water, which could help reduce VOCs. Many—possibly half of all well users—don’t filter their water. The wells ranged in depth from 6 to 1,500 feet, with a median depth of about 140 feet. Of the 2,401 wells studied, 65% had detectable levels of VOCs, and 1% had levels above the EPA maximum contaminant level for the compound(s) observed. The most common compounds found were chloroform, toluene, 1,2,4-trimethylbenzene, and perchloroethene.

Factors associated with the presence of VOCs were dissolved oxygen content, precipitation, the presence of a hazardous waste site within 1 km of the well, aquifer type, and water temperature. The authors note that identifying factors associated with VOC occurrence may aid in understanding the sources, transport, and fate of these compounds in groundwater.

## Figures and Tables

**Figure:**
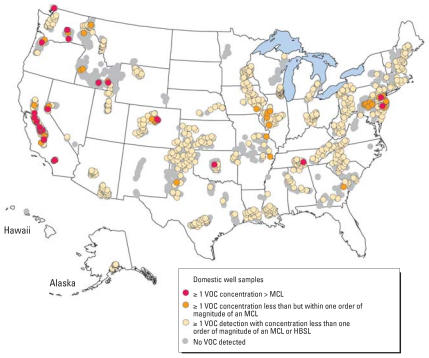
Of 2,401 wells assayed by Rowe et al., 1% had water containing VOCs above EPA maximum contaminant levels.

